# Kawasaki disease − often a diagnostic dilemma in pediatric population: a case report

**DOI:** 10.1097/MS9.0000000000000472

**Published:** 2023-04-07

**Authors:** Subash Subedi, Swikriti Shrestha, Sujata Khatri Chhetri, Sandesh Lamichhane, Swastika Dhakal, Pravakar Dahal, Shubha Baniya, Santosh Pokhrel

**Affiliations:** a Chitwan Medical College Teaching Hospital, Chitwan; b Gandaki Medical College Teaching Hospital and Research Center, Pokhara; c Dhading Hospital, Dhading; d Kalika Municipal Hospital, Chitwan; e Fewa City Hospital, Pokhara, Nepal

**Keywords:** Kawasaki disease, vasculitis, nonresolving fever, case report

## Abstract

Kawasaki disease (KD) is the most common cause of childhood vasculitis affecting small- and medium-sized arteries. The cause of this disease is unknown with an overall prevalence of 0.10% making this a rare entity.

**Case presentation:**

Here the authors present an index case of a 2-year-old child presenting with a persistent high-grade fever of more than 5 days and a 3-day history of bilateral swelling of hands and feet along with cervical lymphadenopathy. On the subsequent day of admission, the child developed mucocutaneous symptoms and cervical lymphadenopathy. The diagnosis of KD was made, and it was successfully treated with intravenous immunoglobulin and aspirin.

**Clinical discussion:**

Timely diagnosis and early treatment of KD are challenging due to the lack of definitive diagnostic tests. Watchful waiting for symptoms may be necessary before a diagnosis can be made because not all clinical symptoms are present simultaneously as in the index case.

**Conclusions:**

This case highlights considering KD as a differential diagnosis of nonresolving fever in children with mucocutaneous findings. Intravenous immunoglobulin along with aspirin is the mainstay of therapy and should be started as early as possible to prevent detrimental cardiac complications. There is a high tendency of diagnostic dilemmas due to a wide array of nonspecific presentations thus healthcare providers must be more vigilant of this entity.

## Introduction

Highlights

- Kawasaki disease is an acute, self-limiting vasculitis that occurs predominantly in children.
- There is a high tendency of disease misdiagnosis due to a wide array of nonspecific presentations thus leading to detrimental cardiac complications.
- Intravenous immunoglobulin along with aspirin is the mainstay of therapy and should be started within the first 10 days of fever onset.

Kawasaki disease (KD) is an acute, self-limiting vasculitis of small and medium vessels of unknown etiology affecting children of all ages and thus accounts for the majority of the incidence of vasculitis in this population^1^. However, the occurrence of the disease itself is rare with an overall prevalence of 0.10% in Nepal^2^. As the symptoms of the disease are disseminated widely over a period of time, patients can have heterogeneous presentations ranging from fever without any localizing symptoms to fever with an array of mucocutaneous symptoms. Consequently, a greater number of patients initially present with characteristics insufficient to meet the criteria needed to diagnose KD impose the risk of misdiagnosis and delay in specific treatment^3^. Herein, we present a case of a 2-year-old child with high-grade fever for 5 days without localizing signs but mucocutaneous manifestations were detected later in the course of hospitalization, making the diagnosis of classic KD imminent. The case has been documented as per the SCARE 2020 guidelines^4^.

## Case presentation

**Figure 1:**
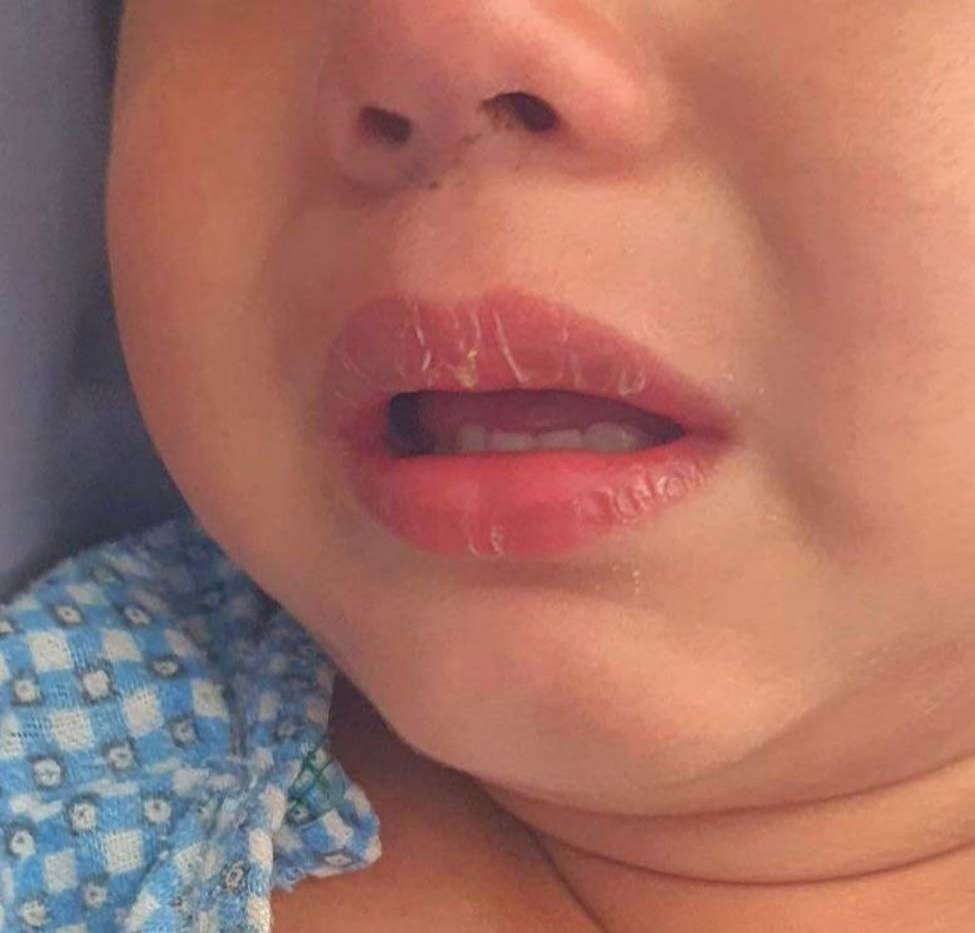
Picture showing cracked lips.

**Figure 2:**
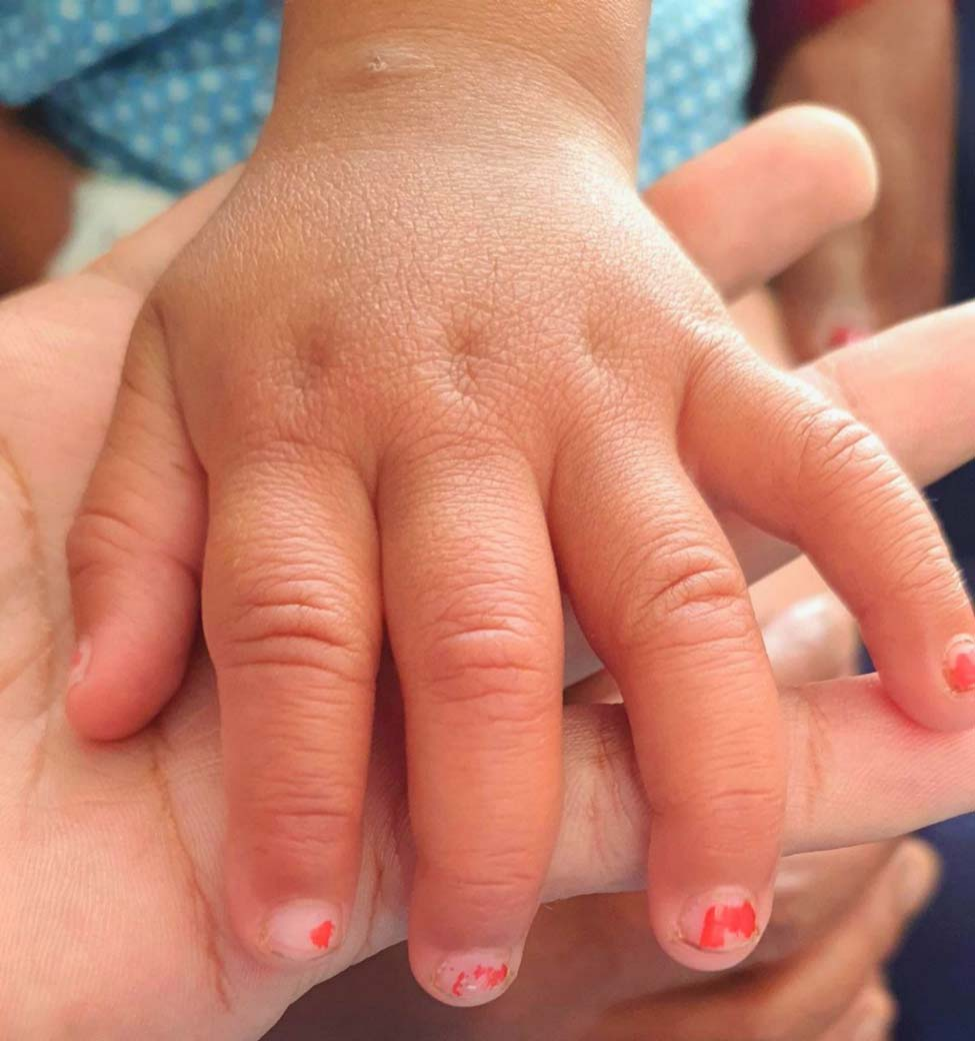
Picture showing edematous hands.

A previously healthy 2 years boy presented to the Department of Emergency Medicine with a 5-day history of persistent high-grade fever with a maximum recorded temperature of 103°F not relieving on antipyretics and a 3-day history of bilateral swelling of hands (Fig. 1) and feet. There was no significant past medical history and no history of drug allergy. Family history was insignificant. On admission, physical examination was remarkable for fever of 102°F, tachypnea, tachycardia, erythematous cracked lips (Fig. 2), bilateral erythema and edema of extremities, and unilateral palpable nontender cervical lymph nodes. Systemic examinations were benign.

His laboratory tests showed hemoglobin (Hb) (9.4 g%), white blood cell count (14 830 per mm^3^) (neutrophils 78%), platelet count (302 000 per mm^3^), alanine transaminase (107 U/l), and aspartate transaminase (72 U/l). Serology for scrub typhus and dengue was negative, ruling out the tropical disease. Serum procalcitonin (1.19 ng/ml) was elevated suggesting an infectious source for which a blood culture was sent. A routine urine examination showed many pus cells but no growth in the urine culture. The blood culture showed no growth after 72 h.

**Figure 3:**
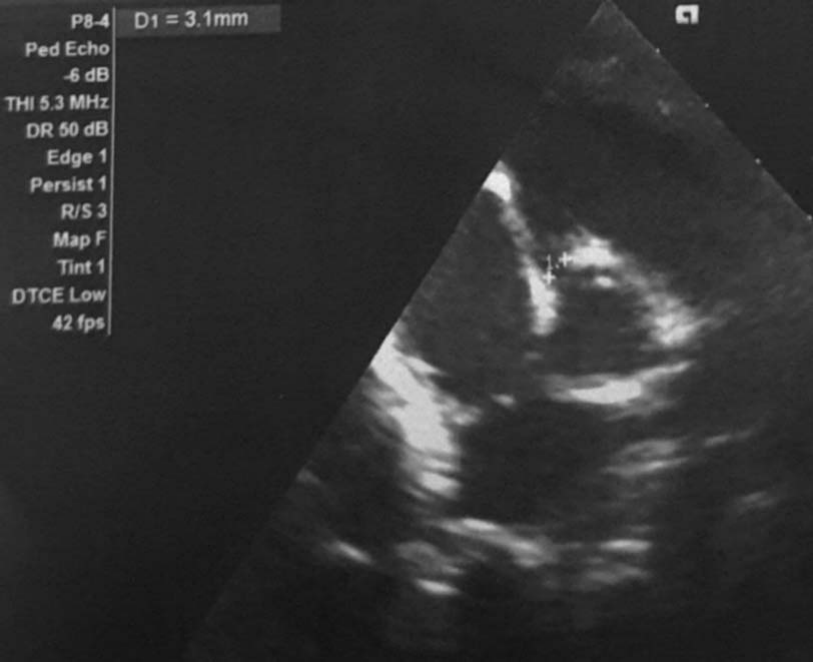
Transthoracic echocardiography showed dilation of the right coronary artery of 3.1 mm with a *z*-score of 4.05.

Initially, the child was treated with empiric intravenous antibiotics in addition to supportive management for suspected bacteremia. He developed multiple spikes of fever (maximum of 102°F) despite the treatment. On the second day of admission, the child was notable for bilateral nonexudative conjunctival injection and nonpruritic, erythematous maculopapular rash on the lateral aspect of the thigh and forearm. Serum concentrations of acute phase reactants, including erythrocyte sedimentation rate (89 mm/h) and C-reactive protein (190 mg/l) were remarkably high. Thus, the diagnosis of classic KD was made based on clinical history and physical examination findings [according to American Heart Association (AHA) criteria]. The absence of various features such as exudative conjunctivitis (e.g. adenovirus), generalized lymphadenopathy (e.g. Epstein–Barr virus), diffuse erythematous rash associated with pharyngitis (e.g. scarlet fever), etc., ruled out the possible mimics of the KD. On further evaluation, transthoracic echocardiography showed dilation of the right coronary artery of 3.1 mm with a *z*-score of 4.05 (Fig. 3) and the left main coronary artery of 2.7 mm with a *z*-score of 2.33.

Intravenous immunoglobulin (IVIG) 2 g/kg and high-dose aspirin (80 mg/kg/day) were administered. The fever and rash subsided over the next 24 h, and serum C-reactive protein levels decreased on subsequent tests (Table 1). The child was discharged after 48 h on low-dose aspirin and advised for 2 weekly follow-ups for echocardiography. After subsequent follow-ups, the echocardiography findings had improved and no complications have been noticed. The patient had good adherence to the medication.

**Table 1:** Timeline of the case.

Day of admission	Presentation	Diagnostic testing	Interventions
First day	Fever for 5 days Vomiting for 3 days Bilateral swelling of hands and feet for 3 days Admitted to PICU	Dengue (NS1 Ag, IgG Ab, IgM Ab): negative Antiscrub typhus IgM: negative Urine RE/ME: plenty of pus cell Procalcitonin: 1.19 ng/ml Hb: 9.4 g/dl WBC: 14 830 per mm^3^ SGPT/ALT: 107 U/l SGOT/AST: 72 U/l	IV Ceftriaxone IV PCM IV fluids
Second day	Persistent fever newly developed maculopapular rash on thighs and forearm Bilateral nonexudative conjunctival injection Diagnosis of KD based on AHA criteria	CRP: 190 mg/l ESR: 89 mm/h Dilated coronaries on ECHO	IVIG 2 g/kg High-dose aspirin (80 mg/kg/day)
Third day	Two spikes of fever and gradually subsided	Urine and blood culture: no growth	IV PCM Cold sponging
Fifth day	Afebrile	CRP: 72.2 mg/l	Switched to low-dose aspirin 5 mg/kg/day
Sixth day	Afebrile		Discharged on low-dose aspirin Advised follow-up for echocardiographic screening

AHA, American Heart Association; ALT, alanine transaminase; AST, aspartate transaminase; CRP, C-reactive protein; ESR, erythrocyte sedimentation rate; Hb, hemoglobin; Ig, immunoglobulin; IV, intravenous; KD, Kawasaki disease; PCM, Paracetamol; WBC, white blood cell.

## Discussion

KD is an acute, self-limiting vasculitis of unknown etiology that occurs predominantly in infants and young children and is a major cause of acquired cardiac disease in children^5^. Timely diagnosis and early treatment of KD are challenging due to the lack of definitive diagnostic tests that could represent an obstacle to reducing the incidence of cardiovascular involvement^6^.

Classic KD is diagnosed based on clinical criteria which include fever for at least 5 days along with at least four of the following principal clinical features: erythema and cracking of lips; bilateral bulbar conjunctival injection without exudates; polymorphous rash; erythema and edema of hands and feet; and cervical lymphadenopathy greater than 15 mm in diameter, usually unilateral^5^. Furthermore, coronary artery aneurysm (CAA) in the setting of compatible febrile illness is pathognomonic for KD which is also the most notable cardiac complication of this disease^7^. The manifestation of this entity ranges from no apparent involvement of the coronary artery to the presence of a life-threatening aneurysm accounting for ~15–20% of the cases^8^. The index case had a small aneurysm (*z*-score 4.05 in right coronary artery) as per the classification based on the *z*-score^9^.

Watchful waiting for symptoms may be necessary before a diagnosis can be made because not all clinical symptoms are present simultaneously^5^. Our case presented with fever along with erythematous cracked lips, bilateral erythema and edema of extremities, and unilateral palpable cervical lymph nodes but the occurrence of nonexudative conjunctival injection and diffuse maculopapular eruption/rash on the second day of admission (6 days after onset of fever) against the background of unresolved fever despite antipyretic and antibiotic therapy led to the establishment of the KD diagnosis based on AHA criteria^5^. Elevated white blood cell count and platelet count, transaminases and acute phase reactants, anemia and sterile pyuria in the index child are surrogate laboratory findings supporting KD.

IVIG along with aspirin is the mainstay of therapy and should be started within the first 10 days of fever onset. IVIG is given at 2 g/kg in a single infusion and aspirin is given at 80–100 mg/kg/day during the acute phase of the disease and the dose is usually reduced after the child has been afebrile for 48–78 h^5^. The frequency of CAA and its associated mortality has been significantly decreased due to the timely administration of IVIG therapy^10^. Studies have demonstrated that treatment with IVIG within 10 days of illness drastically diminishes the risk of CAA by 5-fold compared with those not treated with IVIG. Thus, it is quite evident from the existing literature that the delay in providing adequate treatment may lead to fatal complications^11^,^12^.

Because the diagnosis of classic KD is based solely on nonspecific clinical presentations, clinicians become skeptical when it comes to distinguishing KD from bacterial and viral infections. The presence of the incomplete image at the presentation further delays the identification of KD. The prevalence of KD is 0.1% according to a study conducted in Nepal^2^, and this disease may be underdiagnosed because pediatricians and dermatologists are not familiar with this entity.

## Conclusions

Kawasaki is self-limiting vasculitis manifesting with a dreadful complication if not treated timely. A high index of suspicion is required in cases with nonresolving fever and the correlation of clinical manifestations is crucial to obtain an early diagnosis of KD. Prompt treatment with IVIG should be instituted as early as possible to reduce the inevitable complications associated with KD.

## Ethical approval

This case report did not require review by the institutional review committee.

## Consent

Written informed consent was obtained from the patient for the publication of this case report and accompanying images. A copy of the written consent is available for review by the Editor-in-Chief of this journal on request.

## Sources of funding

The authors declare that this work was not supported by any grants.

## Authors contributions

S.S.: concept of the study, collection of data, literature review, and writing the manuscript. S.S. and S.K.C.: literature review and writing the manuscript. S.L., S.D., P.D., S.B., S.P.: literature review, revising, and editing the manuscript. All authors were involved in drafting and revising the manuscript and approved the final version.

## Conflicts of interest disclosure

All the authors declare that they have no competing interests.

## Research registration unique identifying number (UIN)

NA.

## Guarantor

Sandesh Lamichhane, Chitwan Medical College Teaching Hospital, Chitwan, Nepal. E-mail: doc.lamichhane@gmail.com

## Provenance and peer review

Not commissioned, externally peer-reviewed.
